# VirulenceFinder for *Enterococcus faecium* and *Enterococcus lactis*: an enhanced database for detection of putative virulence markers by using whole-genome sequencing data

**DOI:** 10.1128/spectrum.03724-23

**Published:** 2024-02-08

**Authors:** Louise Roer, Hülya Kaya, Ana P. Tedim, Carla Novais, Teresa M. Coque, Frank M. Aarestrup, Luísa Peixe, Henrik Hasman, Anette M. Hammerum, Ana R. Freitas, On behalf of the ESCMID Study Group for Epidemiological Markers (ESGEM)

**Affiliations:** 1 Department of Bacteria, Parasites and Fungi, Statens Serum Institut, Copenhagen, Denmark; 2 Group for Biomedical Research in Sepsis (BioSepsis), Instituto de Investigación Biomédica de Salamanca, Salamanca, Spain; 3 Grupo de Investigación Biomédica en Sepsis-BioSepsis, Hospital Universitario Río Hortega, Instituto de Investigación Biomédica de Salamanca (IBSAL), Valladollid, Spain; 4 UCIBIO, Departamento de Ciências Biológicas, Laboratório de Microbiologia, Faculdade de Farmácia, Universidade do Porto, Porto, Portugal; 5 Associate Laboratory i4HB, Faculty of Pharmacy, University of Porto, Institute for Health and Bioeconomy, Porto, Portugal; 6 Department of Microbiology, Ramón y Cajal University Hospital and Ramón y Cajal Health Research Institute (IRYCIS), Madrid, Spain; 7 Network Research Centre for Infectious Diseases (CIBERINFEC), Instituto de Salud Carlos III, Madrid, Spain; 8 Research Group for Genomic Epidemiology, Technical University of Denmark, National Food Institute, Lyngby, Denmark; 9 1H-TOXRUN—One Health Toxicology Research Unit, University Institute of Health Sciences, CESPU, CRL, Gandra, Portugal; Quest Diagnostics Nichols Institute, San Juan Capistrano, California, USA

**Keywords:** virulence, PVM, web tool, WGS

## Abstract

*Enterococcus faecium* (*Efm*) is a leading cause of hospital-associated (HA) infections, often enriched in putative virulence markers (PVMs). Recently, the *Efm* clade B was assigned as *Enterococcus lactis* (*Elts*), which usually lack HA-*Efm* infection markers. Available databases for extracting PVM are incomplete and/or present an intermix of genes from *Efm* and *Enterococcus faecalis*, with distinct virulence profiles. In this study, we constructed a new database containing 27 PVMs [*acm, scm, sgrA, ecbA, fnm, sagA, hylEfm, ptsD, orf1481, fms15, fms21-fms20* (pili gene cluster 1, PGC-1), *fms14-fms17-fms13* (PGC-2), *empA-empB-empC* (PGC-3), *fms11-fms19-fms16* (PGC-4), *ccpA, bepA, gls20-glsB1,* and *gls33-glsB*] from nine reference genomes (seven *Efm* + two *Elts*). The database was validated against these reference genomes and further evaluated using a collection of well-characterized *Efm* (*n* = 43) and *Elts* (*n* = 7) control strains, by assessing PVM presence/absence and its variants together with a genomic phylogeny constructed as single-nucleotide polymorphisms. We found a high concordance between the phylogeny and *in silico* findings of the PVM, with *Elts* clustering separately and mostly carrying *Elts-*specific PVM gene variants. Based on our validation results, we recommend using the database with raw reads instead of assemblies to avoid missing gene variants. This newly constructed database of 27 PVMs will enable a more comprehensive characterization of *Efm* and *Elts* based on WGS data. The developed database exhibits scalability and boasts a range of applications in public health, including diagnostics, outbreak investigations, and epidemiological studies. It can be further used in risk assessment for distinguishing between safe and unsafe enterococci.

**IMPORTANCE:**

The newly constructed database, consisting of 27 putative virulence markers, is highly scalable and serves as a valuable resource for the comprehensive characterization of these closely related species using WGS data. It holds significant potential for various public health applications, including hospital outbreak investigations, surveillance, and risk assessment for probiotics and feed additives.

## INTRODUCTION

*Enterococcus faecium* (*Efm*) is one of the most common causes of hospital-associated (HA) infections and is among the most difficult-to-treat organisms (1–3). Traditionally, phylogenetic analysis of *Efm* has supported the species split into two major clades: clade A, which includes most isolates associated with hospitalized humans, and clade B, which includes isolates from community-associated (CA) individuals. However, recent research has reassigned clade B as *Enterococcus lactis* (*Elts*) species, which generally does not contain the markers present in *Efm* isolates of clade A (4). This implies that the population structure of *Efm* is currently exclusively represented by what is referred to as clade A. Clade A further subdivides into A1, primarily comprising clinical isolates, and A2, predominantly consisting of animal-associated isolates. Notably, the classification of animal isolates has been a subject of recent controversy, as these isolates have demonstrated the tendency to form multiple distinct clusters (5). Nonetheless, human A2 carriage is primarily observed in the gut of individuals from the community rather than hospital patients (5–7). When compared to CA *Efm* isolates or *Elts*, HA-*Efm* are clearly associated with increased presence, differential expression, or functional forms of genes encoding a variety of putative or confirmed virulence determinants, enabling them to thrive in the harsh environment of hospitals (8). HA-*Efm* are often enriched (>25 genes) in a plethora of putative virulence markers (PVMs) including many surface proteins, pili, and secreted virulence factors relevant to the adhesion to host tissues, biofilm production, and pathogenesis (8–10). Whole-genome sequencing (WGS) has provided a better understanding of the core and accessory genomes of commensal and outbreak *Efm* isolates and unraveled several novel PVM in recent years (11–16). Nevertheless, they are often arbitrarily selected to be tested in epidemiological studies, thus precluding harmonized correlations to infer a pathogenic potential. Moreover, currently available web tools to extract PVM from WGS data [VirulenceFinder, Center for Genomic Epidemiology (CGE), www.genomicepidemiology.org, and virulence factor database (VFDB), http://www.mgc.ac.cn/VFs/] are incomplete and intermix genes from *Efm* and *Enterococcus faecalis* that have clear distinct virulence profiles. Finally, the nomenclature of several virulence markers is often confusing (especially for surface genes), and merely assessing the presence/absence of specific genes may not always be sufficient (e.g., pili genes) (9, 11).

Currently, the CGE provides the publicly available, user-friendly web tool VirulenceFinder, which enables the identification of 26 virulence genes in WGS data of enterococci. This database includes only the most important gene markers, 5 from *Efm* and 22 from *E. faecalis*, known in 2016, when the database was constructed. Nevertheless, it does not account for the significant differences between species, gene sequences, and the vast body of new knowledge generated through the analysis of WGS data in PVM described afterward in *Efm* species in particular.

One of the most comprehensive studies on this topic analyzed the distribution of 33 PVMs in a set of 328 diverse *Efm* isolates (clinical, healthy humans, food-producing animals, foodstuffs, wild birds, and aquatic environments) obtained during 1986–2015, by PCR-based assays and sequencing (9). Freitas et al. described that 21 out of the 33 PVMs were strain-/clade-specific. Building on these findings, we selected these 21 genes and included six additional PVMs that were described after that project, making a total of 27 genes that have been experimentally tested in *Efm* (or *Elts*) to create a novel and robust database that should be updated along the growing knowledge of new PVM. Given the genomic proximity between *Efm* and *Elts* in comparison to other enterococcal species*,* we aimed to shape a user-friendly web tool for the detection and extraction of virulence genes from genomes representing both species.

## MATERIALS AND METHODS

### Selection of virulence markers and construction of *Efm-Elts* virulence gene database

Based on the results from Freitas et al. (9), 21 out of the 33 genes that were significantly more associated with human infections and epidemic scenarios (but not exclusive) were selected for the database. In addition to the 21 genes selected from the study by Freitas et al. (9), we have incorporated an additional six genes [*ccpA* (15)*, bepA* (16)*, gls20-glsB1,* and *gls33-glsB* (17)] that were rarely investigated at the time of the first project. In total, our database includes 27 PVMs that have been experimentally tested in *Efm* or *Elts* and have been shown to be differentially distributed between clades or hosts. The genes are involved in different cellular functions and correspond to those (i) encoding cell wall-anchored proteins involved in surface adhesion to the extracellular matrix (*acm, scm, fnm, sgrA, ecbA, fms15,* and four pili gene clusters); (ii) with a role in carbohydrate metabolism and cell growth (*orf1481, ccpA*); (iii) encoding phosphotransferase systems (PTSs) important in carbohydrate transport (*ptsD, bepA*); (iv) that are secreted extracellularly with putative roles in cell growth, biofilm formation (*sagA*), and colonization loads (*hyl*_Efm_); and (v) encoding general stress proteins important in bile salt tolerance and general intestinal adaptation (*gls*-like). Despite its relevance in the pathogenesis of *Efm* (18), the *esp* gene was not included in our database (see Results and Discussion for explanation). A description of the 27 PVMs is listed in Table 1. A detailed list of the genes, including reference genomes, position in references, and locus tag are listed in Table S1.

**TABLE 1:** Gene content of the *E. faecium* and *E. lactis* virulence database^*a*^

Gene	Description	Other designations	Variants in the database	Reference
Surface-exposed cell wall-anchored proteins (*E. faecium* surface proteins)				
*acm*	Adhesin of collagen from *E. faecium*	*fms8*	*Efm*-HV, *Efm*-CV, *Elts*-V	(19)
*scm*	Second collagen adhesin of *E. faecium*	*fms10, orf418*	*Efm*-HV, *Efm*-CV, *Elts*-V	(11)
*fnm*	Fibronectin-binding protein of *E. faecium*	*fbpA*	*Efm*-HV, *Efm*-CV, *Elts*-V	(9)
*sgrA*	Serine-glutamate repeat containing protein A	*fms2, orf2351*	*Efm*-HV	(12)
*ecbA*	*E. faecium* collagen binding protein A	*fms18, orf2430*	*Efm*-HV, *Efm*-CV	(11)
*fms15*	*E. faecium* surface protein 15	*orf2514/orf2515*	*Efm*-HV	(11)
PGC-1 cluster [*fms21(pilA)-fms20*]				
*fms21*	*E. faecium* surface protein 21	*pilA, orf1904*	*Efm*-HV, *Efm*-CV, *Elts*-V	(11)
*fms20*	*E. faecium* surface protein 20	*orf1901*	*Efm*-HV, *Efm*-CV, *Elts*-V	(11)
PGC-2 cluster (*fms14-fms17-fms13*)				
*fms14*	*E. faecium* surface protein 14	*orf2010*	*Efm*-HV, *Efm*-CV, *Elts*-V	(11)
*fms17*	*E. faecium* surface protein 17	*orf2009*	*Efm*-HV, *Efm*-CV, *Elts*-V	(11)
*fms13*	*E. faecium* surface protein 13	*orf2008*	*Efm*-V, *Elts*-V	(11)
PGC-3 cluster (*empABC*)				
*empA*	Endocarditis and biofilm-associated pili A	*ebpA_fm_, fms1, orf2571*	*Efm*-HV, *Elts*-V	(11)
*empB*	Endocarditis and biofilm-associated pili B	*ebpB_fm_, fms5, orf2570*	*Efm*-HV, *Efm*-CV, *Elts*-V	(11)
*empC*	Endocarditis and biofilm-associated pili C	*ebpC_fm_, fms9, orf2569*	*Efm*-HV, *Efm*-CV, *Elts*-V	(11)
PGC-4 cluster (*fms11-fms19-fms16*)				
*fms11*	*E. faecium* surface protein 11	*orf903*	*Efm*-HV, *Elts*-V	(11)
*fms19*	*E. faecium* surface protein 19	*orf905*	*Efm*-HV, *Elts*-V	(11)
*fms16*	*E. faecium* surface protein 16	*orf907*	*Efm*-HV, *Elts*-V	(11)
Carbohydrate metabolism and cell growth				
*orf1481*	Sugar binding protein encoded by a genomic island		*Efm*-HV	(20)
*ccpA*	Carbon catabolite control protein A		*Efm*-HV, *Efm*-CV, *Elts*-V	(15)
Phosphotransferase systems				
*ptsD*	Enzyme IID subunit of a phosphotransferase system		*Efm*-HV	(14)
*bepA*	Biofilm and endocarditis-associated permease A/PTS transporter subunit EIIC	*fruA*	*Efm*-HV, *Efm*-CV	(16)
Biofilm and colonization				
*hyl*_Efm_	Glycosyl hydrolase		*Efm*-HV	(21)
*sagA*	Secreted antigen A		*Efm*-HV, *Efm*-CV, *Elts*-V	(20)
General stress proteins				
*glsB1*	General stress protein GlsB1 (*gls20-glsB1* cluster)		*Efm*-V, *Elts*-V	(17)
*gls20*	General stress protein GlsB20 (*gls20-glsB1* cluster)		*Efm*-V, *Elts*-V	(17)
*glsB*	General stress protein GlsB (*gls33-glsB* cluster)		*Efm*-V, *Elts*-V	(17)
*gls33*	General stress protein Gls33 (*gls33-glsB* cluster)		*Efm*-V, *Elts*-V	(17)

^*a*^ *Efm*-HV, *E. faecium* hospital variant; *Efm*-CV, *E. faecium* community variant; *Efm*-V, *E. faecium* variant; *Elts*-V, *E. lactis* variant.

The *Efm-Elts* database was constructed as a FASTA database, comprising a total of 27 PVMs. Each sequence represents a specific gene variant (if existing) representing the *Efm* hospital and *Efm* community clades or *Efm* in general, together with the *Elts* clade. The different variants were assigned based on their comparison with the virulence gene variants previously described by Qin et al. (22) as hospital and community variants extracted from HA and CA genomes, respectively. Some of these genomes were included in this study as HA and CA reference genomes, with some of the latter actually corresponding to *Elts*. Accordingly, *Efm* hospital gene variants (designated as *Efm-*HV here, with an extended description as “*Entfm*-hosp” in the database) were extracted from one of the four clinical *Efm* genomes: *Efm*_DO, E1162, E1636, or TX0082. The *Efm* community gene variants (designated *Efm-*CV here, with an extended description as “*Entfm*-com” in the database) were extracted from one of the three community *Efm* genomes: C59, VE40C2, or E1071. If the *Efm* hospital variant and community variant were identical, the variant was designated as an *Efm* gene variant (*Efm-*V here and as “*Entfm*” in the database). The *Elts* gene variants (*Elts-*V here and as “*Entls*” in the database) were extracted from one of the two *Elts* genomes: TX1330 or 1,141,733 [previously considered as community *Efm* by Qin et al. (22)]. The complete list of genomes with accession numbers is listed in Table S2. For each gene, the coding sequence derived from gene prediction was extracted from the NCBI nucleotide database.

### Sequence data, whole-genome sequencing, and sequence analysis

Paired-end sequence data were either retrieved from the European Nucleotide Archive (https://www.ebi.ac.uk/ena/) or sequenced at Statens Serum Institut (according to Table S3). Genomic DNA was extracted (DNeasy blood and tissue kit; Qiagen, Copenhagen, Denmark) with subsequent library construction (Nextera kit; Illumina, Little Chesterford, UK) and whole-genome sequencing (NextSeq 550; Illumina, Little Chesterford, UK) according to the manufacturer’s instructions, to obtain paired-end reads 2 × 150 bp in length. Raw sequencing data were analyzed using the Bifrost pipeline v. 2.0.8 at Statens Serum Institut (https://github.com/ssi-dk/bifrost) with accepted average coverage of 30× or higher (avg_coverage ≥30). WGS data were either used as raw data or were *de novo* assembled using SKESA v. 2.2 (23), generated with Bifrost. The complete list of control and test genomes is listed in Table S3 with detailed information and accession numbers.

MLST was inferred with the Bifrost pipeline, using the pubMLST database (24). The genomes were further subtyped using the *Efm* cgMLST scheme by de Been et al. in SeqSphere+ v.8.0.1 (Ridom, Münster, Germany), with the suggested threshold of ≤20 allele differences (25). New complex types were registered on the cgMLST Nomenclature Server (https://www.cgmlst.org/ncs).

### Validation of the database and method

To evaluate the genes in the database and validate the method for predicting the presence of the genes, a data set with raw reads and SKESA assemblies of the nine reference genomes was constructed (control samples in Table S3). VirulenceFinder 2.0 at the CGE was designed to use KMA mapping on raw reads and blastn on assemblies (https://cge.food.dtu.dk/services/VirulenceFinder/). Thus, the database was tested with KMA mapping of both raw reads and assemblies against the *Efm-Elts* virulence database, using MyKMAfinder 1.0 (https://bitbucket.org/genomicepidemiology/mykmafinder/src/master/) (26), and blastn on assemblies only, by using MyDbFinder 2.0 (https://bitbucket.org/genomicepidemiology/mydbfinder/src/master/). For validation of the method, the PVM from the corresponding reference should be present at identity (%ID) and coverage (%Cov) of 100%.

### Evaluation of the database

Forty-three *Efm* (17 HA and 26 CA) and seven *Elts* (all CA) isolates presenting different virulence profiles, as obtained by PCR in the study of Freitas et al. (9), were selected for whole-genome sequencing, as described here. These isolates were obtained during 1996–2015 from different sources including hospitalized patients, farm animals, foodstuffs, and the environment (test genomes in Table S3). The 50 test genomes were analyzed for the presence of PVM by mapping the reads against the database, using MyKMAfinder 1.0. A hit with %ID > 95% and %Cov > 80% was considered as the presence of the PVM.

To visualize the presence of PVM in contract to the phylogeny, a phylogenetic analysis was performed on the 50 test genomes, together with the nine control genomes (all listed in Table S3). Single Nucleotide Polymorphisms (SNPs) were identified using the Northern Arizona SNP Pipeline v. 1.2 (27). Briefly, *Efm*_DO with plasmids (CP003583.1, CP003584.1, CP003585.1, and CP003586.1) was used as a reference strain. Duplicate regions of the reference were identified by aligning the reference against itself using NUCmer (28), followed by mapping the raw reads from the test and control genomes against the reference using the Burrows–Wheeler Aligner (29). GATK was used for the identification of SNPs (30).

### Publishing *Efm-Elts* VirulenceFinder on CGE

The *Efm-Elts* virulence database was published on the VirulenceFinder 2.0 web tool (https://cge.food.dtu.dk/services/VirulenceFinder/) as “*Enterococcus faecium* & *Enterococcus lactis,*” enabling a simple and user-friendly tool for the detection of PVM from WGS data. Sequence data can be submitted as either assemblies or raw reads. The tool makes it possible to select the threshold for %ID, describing the threshold of %ID between the input and the best match in the virulence database. Further, it is possible to select a minimum length for the hits to be presented in the output. The output consists of the best-matching PVM from the virulence database and the submitted genome. If the input genome is raw reads, KMA mapping is used to identify the best-matching PVM, whereas blastn is used for assembled genomes. The output contains information on the virulence gene, %ID, length of query, and database gene (template length). If the input data are an assembled genome, information on the contig and position in the contig are provided.

## RESULTS AND DISCUSSION

### Construction and validation of the *Efm-Elts* virulence database

The *Efm-Elts* virulence database was constructed of 27 PVMs, with 22 assigned *Efm* hospital gene variants, 13 *Efm* community gene variants, 5 *Efm* gene variants (same variant represented in both hospital and community *Efm*), and 20 *Elts* gene variants. To evaluate the method and the gene correctness in the database, raw reads and assemblies of the nine control genomes, used for the construction of the database, were tested against the database with two different methods: KMA mapping of raw reads and assemblies against the database and blast of the assemblies against the database (Table 2). From the KMA mapping of the assemblies against the reference PVM, the method correctly assigned the variants in 55 of 65 cases (84.62%), whereas all 65 variants were correctly assigned from the reads (100%). However, for *ccpA,* the *Efm* community gene variant control C59 was reported with an additional hit to the *Efm* hospital gene variant. By assessing the results for C59 (data not shown), the hit to the *Efm* hospital gene variant showed %ID = 97.45% and %Cov = 99.50% with a depth of 18.22×, compared to correct the *Efm* community gene variant with %ID = 100% and %Cov = 100% with a depth of 231.29×. Additionally, when assessing the other hits for C59, the depth was a minimum of 114×, indicating that the extra *ccpA* hit could be due to sequencing errors.

**TABLE 2:** Concordance of PVM in the database and reference genomes

	Total gene variant	No. of control genomes with the virulence gene variant found by		
		KMA mapping		blastn
		Assembly	Reads	Assembly
*Efm* hospital variant	22	20	22	21
*Efm* community variant	13	7	13	13
*Efm* variant^*a*^	5	10	10	10
*Elts* variant	20	18	20	19

^*a*^ For the five genes *fms13, glsB1, gls20, glsB,* and *gls33,* the *Efm* hospital gene variant and community gene variant were 100% identical; thus, only one variant designated *Efm* variant was included in the database. The two references *Efm*_DO (hospital variant) and C59 (community variant) were evaluated against the genes.

By using blastn of the assemblies against the database, the magnitude of errors decreased, and a total of 63 of 65 were correctly assigned (96.92%) for assemblies, indicating that using the current VirulenceFinder tool at CGE would be suitable for our current database, but to ensure the most accurate results in assigning the correct variants of PVMs, it is recommended to use raw reads instead of assembled genomes.

By comparing the newly constructed database with the old version, it becomes evident that the updated database represents a substantial enhancement. The original database comprised a total of 26 genes, with only 5 featuring *Efm* variants (*acm, efaAfm, fsrB, hylEfm*, and *espfm*), while 22 genes were associated with *E. faecalis* species. In contrast, in the new database, all 27 genes have been described as *Efm* variants (either *Efm, Efm-*HA, or *Efm-*CA variants). Notably, the foundation for this enhancement was laid in the study by Freitas et al. (9), where three of the five *Efm* genes in the original database (*acm, hylEfm,* and *espfm*) were thoroughly investigated.

The *esp* (*Enterococcus* surface protein) gene is known for its crucial role in biofilm formation and correlation with diseases. *Esp* possesses a highly repetitive structure (31). When searching for *esp* from *Efm* in the UniProt database (http://www.unipro.org) on 20 February 2023, we obtained 17 results for the gene, with lengths ranging from 112 to 1,975 amino acids. Given the considerable diversity in both size and structure of this gene, it proves challenging to select representatives for the various variants. Based on this, we decided not to include *esp* in this current version of the database.

The *efaAfm* gene has been shown to be widely distributed among *Efm* strains, appearing almost as species-specific, and its role is still not totally understood (32), and for these reasons, it was not included in the previous study by Freitas et al. (9). Based on the limited knowledge of this gene, it is currently not incorporated into the new database. However, as our understanding is growing and new insights emerge, there remains the possibility of incorporating the gene into future iterations of the database.

Regarding the *fsrB* gene, present in the old database, it is a component of the *fsr* locus, which includes three regulatory genes, *fsrA*, *fsrB,* and *fsrC*, located upstream of the *gelE-sprE* operon. These genes are mostly detected in *E. faecalis* (33); however, the old database reports a single detection found in an *Efm* genome, although this specific *Efm* genome is not publicly available (34). Thus, we decided not to include *fsrB* (or the *fsr* operon and *gelE-sprE* operon) in this current version of the database.

### Phylogenetic analysis and evaluation of the database

**Fig 1:**
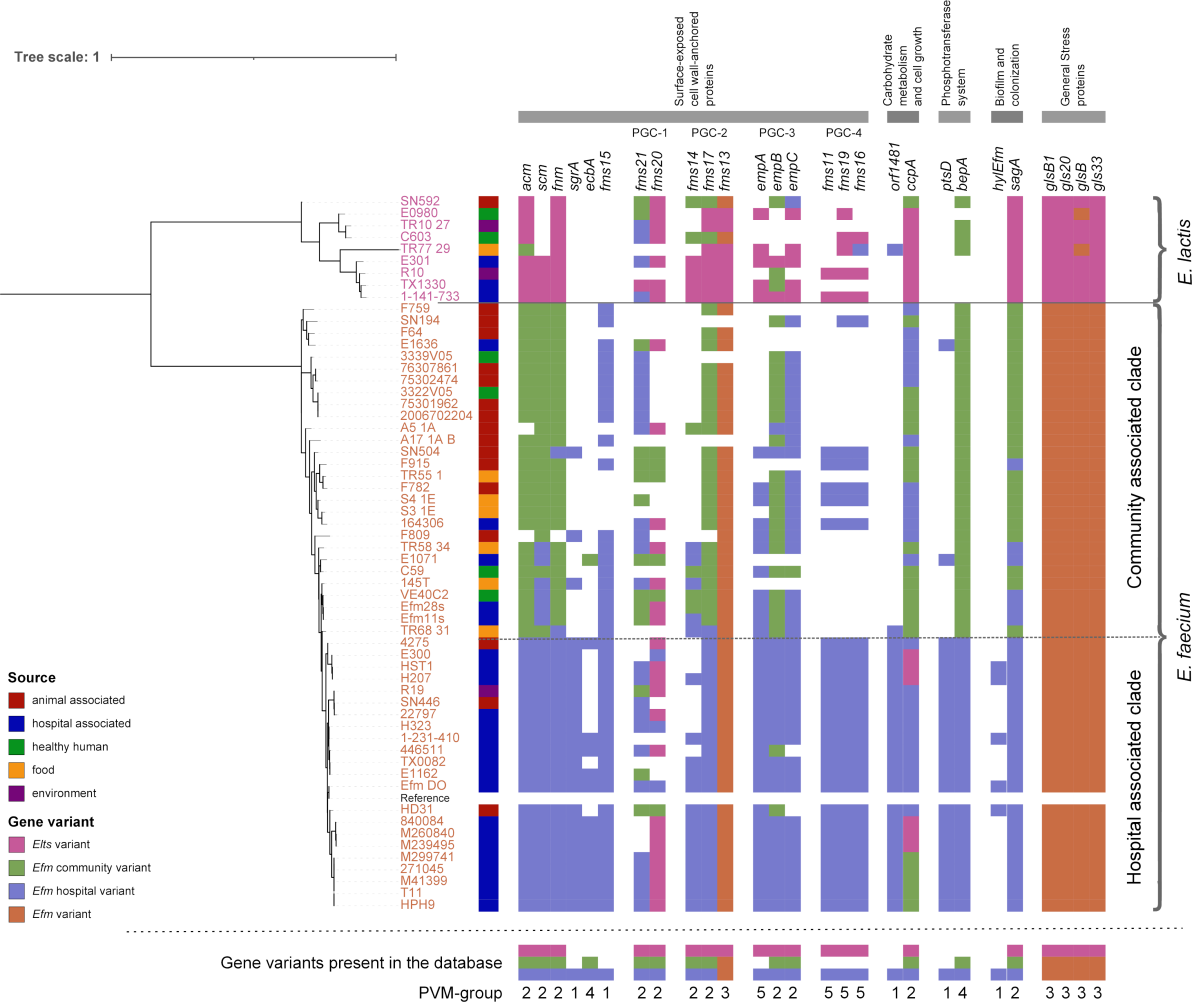
Phylogenetic relationship of 9 *E. lactis* and 50 *E. faecium* genomes. The complete genome of Efm_DO was used as a reference, with the SNP analysis identifying 119,276 SNPs. The best hit from the KMA mapping against the virulence database is indicated by color. The bar at the bottom of the figure illustrates if a given gene variant was present in the virulence database.

To evaluate the performance of the database and the distribution of gene variants, we constructed a phylogenetic tree based on SNP analysis and compared it to the results obtained from the *Efm-Elts* virulence database. From the SNP analysis, 119,276 SNPs were identified between the 59 genomes (50 test genomes and 9 control genomes, Table S3), based on 61.81% of the reference genome, the *Efm*_DO with plasmids. The phylogenetic analysis revealed two distinct clades that clearly separate the *Elts* and *Efm* genomes (Fig. 1).

Assessing the CA clade (generally designated as *Efm* A2) of the *Efm* genomes, most isolates were associated with animals, food, and healthy humans, whereas the HA clade (generally designated as *Efm* A1) is mainly hospital-associated (patients). No community gene variants of *sgrA*, *fms15, empA, fms11, fms19, fms16, hylEfm, orf1481,* and *ptsD* were found among the community reference genomes, thus not included in the database.

Overall, it was notorious from Fig. 1 that HA-*Efm* strains mostly cluster apart and are greatly enriched in *Efm* hospital gene variants (purple, *Efm*-HV) with a few exceptions for *fms21-fms20, empB,* and *ccpA* genes. CA-*Efm* strains from animal, food, and environmental sources contain the greatest admixture of *Efm* community gene variants (green, *Efm-*CV), hospital gene variants (purple, *Efm-*HV), and also *Elts* gene variants (pink, *Elts*-V). As previously observed (9, 10), HA-*Efm* are more enriched in PVM, even though some of the hospital gene variants can be also found among community strains. *Elts* genomes were greatly associated with unique *Elts* gene variants (*Elts-*V in pink), but an intermix of *Elts* gene variants with *Efm-*HV and/or *Efm-*CV occurred in several cases (*acm, fms21, fms14, fms17, empB, empC, fms16, orf1481, bepA,* and *glsB*).

Our results showed the clustering of the 27 PVMs included in the database into five groups of genes according to their distribution in the enterococcal genomes analyzed: PVM-1, gene group represented only by *Efm* hospital gene variants (*Efm*-HV); PVM-2, gene group represented by *Efm* hospital gene variants (*Efm-*HV), *Efm* community gene variants (*Efm-*CV), and *Elts* gene variants (*Elts-*V); PVM-3, gene group represented by a single gene variant in *Efm* and *Elts*; PVM-4, gene group represented by only an *Efm* hospital gene variant (*Efm-*HV) and a community gene variant in *Efm* (*Efm-*CV); and PVM-5, gene group represented by *Efm* hospital gene variants (*Efm*-HV) and *Elts* gene variants (*Elts*-V).

PVM-1 is a gene group represented only by an *Efm* hospital gene variant (*Efm-*HV): *sgrA, fms15, hyl_Efm_, orf1481,* and *ptsD*. Based on the reference genomes included in the study, we were not able to identify an *Efm-*CV or *Elts*-V in this set of genes. Thus, the variants always corresponded to hospital gene variants, even in the rare positive community *Efm* and *Elts* genomes (the exception was *fms15* quite commonly found among *Efm* CA clade). This observation suggests that these genes are significantly linked to HA-*Efm* isolates but can also exchange with community *Efm* strains (e.g., *sgrA*) and even *Elts* (e.g., *orf1481*). As suggested by van Hal et al. (5), the HA-*Efm* clade represents a genetic continuum between the community *Efm* and *Elts* and an ongoing source of genetic determinants for clinical strains. Such miscellaneous networks from CA, including strains from various sources, may be highly dynamic with virulence genes being interchangeable within mobile genetic elements among different clades and also between *Efm* and *Elts* species.

PVM-2 is a gene group presenting *Efm* hospital gene variants (*Efm-*HV), *Efm* community gene variants (*Efm-*CV), and *Elts* gene variants (*Elts-*V): *acm, scm, fms21, fms20, fms14, fms17, empB, empC, sagA, fnm,* and *ccpA*. These genes were found in most isolates, but with variable sequences depending on the origin of the genomes. In most of the cases, HA-*Efm* isolates carried *Efm-*HV whereas CA-*Efm* isolates carried *Efm-*CV and *Elts* carried *Elts-*V, highlighting the importance of considering gene sequences and not just the presence of a particular gene in a given strain. Indeed, differential expression between clinical and community genomes has been previously documented for some of these genes like *sagA* (35). However, there was no consistent clustering of gene variants into the corresponding clade or species in 100% of the cases (expected would be *Efm-*HV in HA clade, *Efm-*CV in CA clade, and *Elts-*V in *Elts* genomes).

When examining the gene variants within the *Elts* genomes, we observed that the *Elts* gene variants were well represented whenever they were present in the database. However, in the case of the gene *fms21*, the *Elts* gene variant was only found in the reference genome and was absent in all other *Elts* genomes. When assessing the present variants of *fms21* across all genomes, the *Efm* community and hospital gene variants are found among all different sources (animals, hospital-associated, and healthy humans), indicating that *fms21*, the major pilus protein of this cluster, is not highly conserved in one group or the other. In contrast, *fms20* [also belonging to pili gene cluster 1 (PGC-1)] mainly hits to the *Elts* variant across the entire data set, suggesting a hypothetical origin of this gene in particular from *Elts* species. The most used reference for the community variant, C59, did not carry either of the two genes from PGC-1. Previous studies suggested that the *fms21-fms20* gene cluster has probably originated in ancestral and commensal isolates on large transferable plasmids explaining the lack of significant differences in its occurrence between HA and CA clades (36, 37).

PVM-3 is a gene group represented by an *Efm* gene variant and an *Elts* gene variant: *fms13*, *glsB*-like genes. Within the PGC-2 gene cluster (*fms14-fm17-fms13*), *fms13*, which is the major pilus protein from this cluster, was the only gene that was not represented by an *Efm* hospital and community variant. However, it is important to note that this finding does not necessarily imply the absence of a community variant for this gene. In our current database, all community reference and test genomes carried *fms13* genes with at least 95% identity to that of the *Efm*_DO reference clinical genome. As the database continues to grow and will be updated, the presence of community variants for *fms13* might be found and confirmed in an *Efm* community reference genome.

Assessing the general stress proteins, the four *glsB*-like genes were present in all genomes. Except for two *Elts* genomes (that mapped to *Efm*-V of *glsB*), all *Elts* genomes did map to the *Elts-*V, and all *Efm* genomes did map to the *Efm-*V. Thus, at least *glsB1, gls20,* and *gls33* genes could be suggested as markers to differentiate *Elts* and *Efm*.

PVM-4 is a gene group represented by only an *Efm* hospital gene variant (*Efm-*HV) and an *Efm* community gene variant (*Efm-*CV): *ecbA* and *bepA*. For both these genes, the lack of intermixing of variants between the HA clade (all with *Efm-*HV variants) and the *Efm* CA clade or *Elts* genomes was notable. Thus, this corresponds to the only cases where the hospital gene variant was found to be confined to isolates in the HA clade, which corroborates previous findings (16). All *Elts* genomes that were positive for *bepA* carried variants with 97.82% identity to the *Efm-*CV, indicating a possible *bepA Elts*-V. To extract the *Elts*-V, additional reference genomes (complete and annotated genomes) should be identified in the future to verify the gene and expand the database.

PVM-5 is a gene group represented by an *Efm* hospital gene variant (*Efm-*HV) and an *Elts* gene variant: *empA, fms11, fms19,* and *fms16*. With a unique exception for *fms16*, all *Efm* (from both HA and CA clades) carried hospital gene variants, and all *Elts* carried *Elts* gene variants of these four genes. The exception corresponded to an *Elts* isolate carrying an *Efm* hospital gene variant of the *fms16* gene (Fig. 1). In this specific case, it is likely that our database will benefit from future analyses of additional genomes. These analyses can help determine the potential existence of a community gene variant for these genes in *Efm*, especially since none were identified in any of the community reference genomes used in this study.

Considering all results, the presence of PVMs like *ptsD, orf1481,* and *sgrA* and the hospital variants of the pili gene clusters (PGC-1 to 4) was identified as good markers for hospital-associated *Efm* strains. However, assessing Fig. 1, hospital gene variants for the PGC-1 and PGC-3 seem to be highly represented among the community strains, whereas PGC-2 community gene variants seem to be more correlated to community sources, and PGC-4 only seems to be present in few community strains. Thus, *ptsD, orf1481,* and *sgrA* and the hospital variants of PGC-2 and PGC-4 seem to be good markers for the hospital-associated clade. Looking carefully at Fig. 1, complete pili gene clusters comprising only hospital gene variants are only observed within HA-*Efm* strains. One exception is for PGC-3 and five cases of PGC-4, for which complete hospital gene variants of the pili gene clusters were found in *Efm* community-associated isolates. Nevertheless, as previously documented (9), the presence of PGC-4 has been infrequently observed outside the *Efm-*HA clade. When encountered, it tends to be associated with multidrug-resistant vancomycin-resistant *Enterococcus* and/or ampicillin-resistant strains found in both human and animal populations. Considering the main differences between *Efm* and *Elts* species, among the 27 genes examined, only *ptsD, hylEfm, sgrA, ecbA,* and *fms15* were found exclusively in *Efm*.

Finally, it is important to emphasize that the outcomes may vary depending on the collection used. We cannot rule out the possibility of a selection bias in the description of our results using this collection in particular. That is why it is crucial to continuously update the database as new results emerge from different projects. New potential virulence markers can be submitted to curators, who will verify the genes and, if relevant, add them to the database.

### Conclusion

This study shows that *Efm-Elts* VirulenceFinder is able to extract putative virulence markers from uploaded WGS data in a reliable and user-friendly manner, which makes it globally accessible to non-bioinformatics users. Our validation analysis revealed that *in silico* typing using raw read data yields more accurate results than using assembled genomes. However, users of the database must be cautious, when interpreting the results in particular concerning the differentiation between hospital and community variants, since it may need to be updated as more data become available. From a public health perspective, we clearly demonstrate that all *Efm* clades or *Efm* and *Elts* share common putative virulence gene variants. In this study, specific PVMs (*ptsD, hylEfm, sgrA, ecbA,* and *fms15*) were not detected in *Elts*. In addition, *ptsD, orf1481,* and *sgrA* and the PGC-2 and PGC-4 (if hospital variants are confirmed) were revealed as good markers for the *Efm* hospital-associated clade. The newly constructed database of 27 PVMs is easily scalable and provides a valuable resource for a comprehensive characterization of *Efm* and *Elts* using WGS data. It can aid in various public health applications, such as hospital outbreak investigations, surveillance, and risk assessment of probiotics (38), and feed additives facilitating the identification and tracking of potentially pathogenic strains.

## Data Availability

Accession numbers for the whole-genome sequences included in this study can be found in Table S3. The new *Efm* and *Elts* VirulenceFinder database (virulence_entfm_entls.fsa) can be downloaded from https://bitbucket.org/genomicepidemiology/virulencefinder_db/src/master/.

## AFTER EPUB

*[This article was published on 8 February 2024 with incorrect information in the funding table. The table was updated in the current version, posted on 14 February 2024.]*

## SUPPLEMENTAL MATERIAL

Supplemental Table S1 spectrum.03724-23-s0001.xlsx Gene content of the *E. faecium* and *E. lactis* virulence database.

Supplemental Table S2 spectrum.03724-23-s0002.xlsx Overview of reference genomes used to construct the *Efm-Elts* VirulenceFinder database.

Supplemental Table S3 spectrum.03724-23-s0003.xlsx Epidemiological background of the 9 Control- and 50 Test- *Enterococcus faecium* and *Enterococcus lactis* genomes analyzed in the present study.

ASM does not own the copyrights to Supplemental Material that may be linked to, or accessed through, an article. The authors have granted ASM a non-exclusive, world-wide license to publish the Supplemental Material files. Please contact the corresponding author directly for reuse.
